# Learning from the Italian experience in coping with COVID-19

**DOI:** 10.1590/0037-8682-0199-2020

**Published:** 2020-06-03

**Authors:** Anna Cristina Calçada Carvalho, Afrânio Kritski

**Affiliations:** 1 Fundação Oswaldo Cruz, Instituto Oswaldo Cruz, Laboratório de Inovações em Terapias, Ensino e Bioprodutos, Rio de Janeiro, RJ, Brasil.; 2 Universidade Federal do Rio de Janeiro, Faculdade de Medicina, Programa Acadêmico de Tuberculose, Rio de Janeiro, RJ, Brasil.

**Keywords:** COVID-19, SARS-CoV-2, Italy, Brazil, Pandemic

## Abstract

**INTRODUCTION:**

In March 2020, the rapid increase in COVID-19 cases overburdened the Italian health system, with the country becoming the pandemic’s epicenter.

**METHODS:**

We present a narrative review based on manuscripts, official documents, and newspaper articles regarding COVID-19 in Italy.

**RESULTS:**

Characteristics of the epidemic, possible causes for its worsening, and the measures adopted across Italian regions are presented.

**CONCLUSIONS:**

In the early stages of an epidemic, effective decision-making is essential to contain the number of cases. Medical support for patients and social isolation measures are the most appropriate strategies currently available to reduce the spread and lethality of COVID-19.

In March 2020, the epidemic caused by the novel coronavirus (or SARS-CoV-2) turned into a global pandemic, with Italy leading the tally with regard to the number of COVID-19 cases as well as fatalities. This turn of events raises an important question: what happened in Italy, the first Western country to be hit by COVID-19, for it to become the epicenter of the pandemic? And, most significantly for us, what can we learn from the Italian experience to reduce the impact of the pandemic in Brazil? Here, we present a narrative review of articles published from February to April 2020 in scientific journals, on institutional websites, and in the press in general, referring to the COVID-19 pandemic in Italy.

During the month of March, the press and social networks broadcast shocking images of Italian hospitals-crowded emergency rooms, patients on stretchers in corridors, surgical centers transformed into intensive care units (ICUs), health professionals becoming ill (and dying), 50-70% of hospital beds occupied by patients with COVID-19, and so on. The scarcity of the necessary equipment put doctors in the position of having to make the drastic decision of who would be placed on respirators based on their chances of survival^1^ ^,^ ^2^.

It should be noted that Italy has one of the best public health systems in the world^3^, so attributing the chaos observed in the system to the scarcity of health services is a simplistic response. Given the suddenness of the onslaught of patients with COVID-19 in need of ICU beds, it is unlikely that any health system would have been prepared to manage the situation.

Based on data from May 17, Italy was the third country by number of COVID-19 deaths (31,763), behind the United States of America (85,860) and the United Kingdom (34,466)^4^. Even considering the limitation of the current statistics of lethality (which have in the denominator the number of patients with positive molecular tests, being that the tested ones are mostly inpatients and, therefore, with more severe disease), the fact is that the Italian numbers are scary. One reason immediately raised to justify this high lethality is that Italy has one of the oldest populations in the world, with about 23% of Italians over 65^5^. Advanced age is a risk factor associated with higher mortality from SARS-CoV-2^6^.

Nursing homes for older adults in Italy have become high-risk locations for their residents, who, in addition to old age, have multiple comorbidities; the nursing home setting has witnessed a large number of deaths by COVID-19. The province of Bergamo, for example, recorded more than 600 deaths in 20 days (out of a total of 6,400 available beds), and 40% of the professionals working in these asylums were on sick leave or in isolation^7^. Although not all deaths can be attributed to COVID-19 owing to the lack of diagnostic confirmation, the high number of deaths and the illness of professionals makes this association probable. On March 8, by means of a decree, without any prior scientific evidence, the political leaders of the Lombardy region appealed to those responsible for nursing homes for older adults to admit patients with less severe forms of COVID-19; the motive was to create space in hospitals and ICUs^8^. As expected, most nursing homes did not respond to the call, fearing the spread of the virus to other older adults in these settings, but some did. The consequences of this decision have recently begun to be discussed in the Italian press, and the real impact needs to be evaluated. However, it is certain that older adults, especially the most weakened and confined in institutions such as nursing homes, are the most vulnerable and therefore, must be the most protected, even avoiding contact with their families to reduce the risk of SARS-CoV-2 transmission.

The advanced age of a large part of the Italian population may be one of the causes of the significant number of deaths, but it is not a comprehensive explanation. Political decisions aimed at limiting the spread of the epidemic across the entire territory were slow to be taken. In an article published in the *Harvard Business Review*, Pisano et al.^9^ performed a detailed analysis of political management of the emergency in Italy and discussed the importance of swift political decision-making and effective action based on available information. The authors emphasized that these actions will be all the more effective the more immediate the decision-making is, and politicians should be prepared for the possibility that, if their actions are quick enough to prevent an epidemic from starting, the measures taken may, in retrospect, be considered excessive or even unnecessary. 

The delay in adopting social isolation measures and restricting movement, together with a public event in late February that involved politicians in Milan exchanging handshakes to symbolize that the economy could not stop because of the epidemic, contributed to misinformation about the risks of virus transmission. In the same month, public events brought together thousands of people in Lombardy. Agricultural fairs in Bergamo and Brescia, a soccer match between Atalanta (Bergamo) and Valencia (Spain) for the Champions League in Milan^10^, and even environmental pollution, a chronic problem in a highly industrialized region^11^, were raised as possible causes of the high concentration of cases in Lombardy, where half of the Italian cases of COVID-19 were registered.

In the article by Pisano et al.^9^, the authors compared Lombardy and Veneto, two of the richest Italian regions. Despite the regions’ similar socioeconomic characteristics, the rapid expansion in COVID-19 cases observed in Lombardy was not observed in Veneto. Among the reasons raised by the authors is the difference in the management of probable cases of COVID-19. Both regions adopted the social distancing measures enacted by the Italian central government. However, in Veneto, these actions were broader, and involved the testing of symptomatic as well as asymptomatic people, home isolation of those who tested positive and their families (with testing of their neighbors), and monitoring and protection of health care workers and other professionals in contact with the public (such as supermarket cashiers and pharmacy and security professionals). Another important aspect of the differentiated approach toward COVID-19 adopted by the Veneto region was the emphasis on home care in mild cases or in the early stages of the disease, which placed a lower burden on hospitals, contrary to what happened in Lombardy^8^, where, owing to the large number of hospitalizations, there was greater exposure of patients and health care teams to nosocomial transmission of the novel coronavirus.

The decentralization of the Italian health system also prevented uniform measures from being adopted across the country^9^. Restrictive measures were gradually implemented, with the progressive closure of cities and regions, but these had questionable results in relation to the effective control of the spread of the virus. The closure of northern Italy decreed by the central government made many Italians from southern Italy who lived there return to their cities of origin, taking the virus to other regions of the country. As Pisano et al.^9^ stated, Italy has followed the rapid spread of the virus, instead of preventing it.

In Italy, hospitals receive around 45% of health system funding, which is higher than the Organisation for Economic Co-operation and Development average (38%)^12^. According to Giuseppe Ippolito, Scientific Director of the National Institute for Infectious Diseases Lazzaro Spallanzani in Rome, priority has been given to excellent hospital care over primary care throughout the territory. In a pandemic situation like the one we are experiencing, there is a need for community-centered care and preparation for new epidemics that may still occur through a central system that coordinates the actions to be implemented. As Ippolito states, “A centralized health model is needed for the management of infectious diseases. Without such a system we will not be able to get out of this”^13^.

At the time of writing this article, on May 17, Italy had reported 223,885 cases of COVID-19. However, after so much desolation, the number of deaths, which in one day almost reached the quota of 1,000, began to fall to less than 200^4^, as did the ICU admissions. Restrictive measures, however late, may have prevented the deaths of 38,000 [13,000-84,000] Italians, according to estimates by the Imperial College^14^.

In Brazil, we are in the ascending phase of the pandemic curve, with an exponential progression of cases and, consequently, deaths^15^. In large metropolitan areas, such as Manaus, Fortaleza, Rio de Janeiro, and São Paulo, the majority of ICU beds are occupied. Contradictory messages from political leaders, delay in a uniform and controlled adoption of containment and mitigation measures, and poor preparation of the health system made us, in May 17, 2020, the fourth country by number of confirmed cases of COVID-19, surpassing Italy and Spain. In Brazil, 233,142 cases and 15,633 deaths have been registered so far^16^. Unfortunately, Italy’s mistakes in pandemic management did not serve as a lesson for Brazil.

From the above, and in the absence of proven effective treatments or a preventive vaccine, we must offer supportive treatment to patients and adopt non-pharmacological measures since, at the moment, the best way for each of us to contribute to slowing the spread of SARS-CoV-2 is to avoid contact with other people. Behaving in this way, we will allow time for the preparation of the health system, increasing our chances of receiving adequate medical care and surviving this new virus.
